# Supplementary material for: Estimands: bringing clarity and focus to research questions in clinical trials
Source: BMJ Open. 2022 Jan 3;12(1):e052953. doi: 10.1136/bmjopen-2021-052953 (PMC8724703; doi:10.1136/bmjopen-2021-052953)
Supplement: Author's manuscript [file bmjopen-2021-052953.draft_revisions.pdf]

BMJ Open

BMJ Open is committed to open peer review. As part of this commitment we make the peer review history of every article we publish publicly available.

When an article is published we post the peer reviewers' comments and the authors' responses online. We also post the versions of the paper that were used during peer review. These are the versions that the peer review comments apply to.

The versions of the paper that follow are the versions that were submitted during the peer review process. They are not the versions of record or the final published versions. They should not be cited or distributed as the published version of this manuscript.

BMJ Open is an open access journal and the full, final, typeset and author-corrected version of record of the manuscript is available on our site with no access controls, subscription charges or pay-per-view fees (http://bmjopen.bmj.com).

If you have any questions on BMJ Open's open peer review process please email info.bmjopen@bmj.com

BMJ Open

Estimands: Bringing clarity and focus to research questions in clinical trials

Journal:	*BMJ Open*
Manuscript ID	bmjopen-2021-052953.R1
Article Type:	Communication
Date Submitted by the Author:	22-Jun-2021
Complete List of Authors:	Clark, Timothy; MRC Clinical Trials Unit at UCL, Institute of Clinical Trials & Methodology<br>Kahan, Brennan; MRC Clinical Trials Unit at UCL<br>Phillips, Alan; ICON Clinical Research UK Ltd, Biostatistics;<br>White, Ian; MRC Clinical Trials Unit at UCL, Clinical Trials & Methodology<br>Carpenter, James; MRC Clinical Trials Unit at UCL, London Hub for Trials Methodology Research; London School of Hygiene & Tropical Medicine, Department of Medical Statistics
**Primary Subject Heading**:	Research methods
Secondary Subject Heading:	Communication, Medical education and training, Medical publishing and peer review
Keywords:	Protocols & guidelines < HEALTH SERVICES ADMINISTRATION & MANAGEMENT, MEDICAL EDUCATION & TRAINING, QUALITATIVE RESEARCH

SCHOLARONE™
Manuscripts

For peer review only - http://bmjopen.bmj.com/site/about/guidelines.xhtml

I, the Submitting Author has the right to grant and does grant on behalf of all authors of the Work (as defined in the below author licence), an exclusive licence and/or a non-exclusive licence for contributions from authors who are: i) UK Crown employees; ii) where BMJ has agreed a CC-BY licence shall apply, and/or iii) in accordance with the terms applicable for US Federal Government officers or employees acting as part of their official duties; on a worldwide, perpetual, irrevocable, royalty-free basis to BMJ Publishing Group Ltd ("BMJ") its licensees and where the relevant Journal is co-owned by BMJ to the co-owners of the Journal, to publish the Work in BMJ Open and any other BMJ products and to exploit all rights, as set out in our licence.

The Submitting Author accepts and understands that any supply made under these terms is made by BMJ to the Submitting Author unless you are acting as an employee on behalf of your employer or a postgraduate student of an affiliated institution which is paying any applicable article publishing charge ("APC") for Open Access articles. Where the Submitting Author wishes to make the Work available on an Open Access basis (and intends to pay the relevant APC), the terms of reuse of such Open Access shall be governed by a Creative Commons licence – details of these licences and which Creative Commons licence will apply to this Work are set out in our licence referred to above.

Other than as permitted in any relevant BMJ Author's Self Archiving Policies, I confirm this Work has not been accepted for publication elsewhere, is not being considered for publication elsewhere and does not duplicate material already published. I confirm all authors consent to publication of this Work and authorise the granting of this licence.

For peer review only

Communication

Estimands: Bringing clarity and focus to research questions in clinical trials

Timothy P Clark, honorary associate professor of medical statistics[1], Brennan C Kahan, senior research fellow[1], Alan Phillips, Sr Director, Biostatistics[2], Ian R White, professor of statistical methods for medicine[1], James R Carpenter, professor of medical statistics[1]

Correspondence to: T Clark, **MRC Clinical Trials Unit at UCL,** Institute of Clinical Trials & Methodology, 90 High Holborn, 2nd Floor, London WC1V 6LJ

Tel: +44 (0)20 7670-4700

E-mail: tim.clark@ucl.ac.uk

Keywords: estimands; estimation; research question; treatment effect; post-randomisation events.

Word count: 3,720

[1]MRC Clinical Trials Unit at UCL, Institute of Clinical Trials andMethodology, University College London, London, UK
[2]Innovation Center, ICON Clinical Research, Marlow, UK

For peer review only

Abstract

Precise specification of the research question and associated treatment effect of interest is essential in clinical research, yet recent work shows that they are often incompletely specified. The ICH E9 (R1) Addendum on Estimands and Sensitivity Analysis in Clinical Trials introduces a framework that supports researchers in precisely and transparently specifying the treatment effect they aim to estimate in their clinical trial. In this paper we present practical examples to demonstrate to all researchers involved in clinical trials how estimands can help them to specify the research question, lead to a better understanding of the treatment effect to be estimated and hence increase the probability of success of the trial.

Background

To illustrate the need for estimands, consider some typical wording for the primary objective of a Parkinson's disease (PD) clinical study:

The primary objective of this study is to demonstrate that Drug X is superior to placebo in patients with idiopathic Parkinson's disease with "wearing off" phenomenon.

We are all familiar with such statements, but what do they actually tell us about the treatment effect to be estimated? We know the treatments to be compared, the patient population of interest and that the aim of the study is to show superiority. An experienced researcher in the field would further recognise that these are patients with advanced PD who are currently receiving levodopa and potentially other medications such as dopamine agonists; that these patients have wearing off phenomenon, which means that the effect of levodopa is wearing off between doses, i.e., Parkinson's symptoms such as problems with movement (motor symptoms) return; and finally, that the study is being conducted to see if the addition of Drug X to background medication consisting of levodopa plus another anti-Parkinson drug, e.g. dopamine agonist or MAO-B inhibitor, will improve control of motor symptoms[1].

Post-randomisation events

To fully understand the treatment effect of interest, the objective statement additionally needs to convey what the outcome variable (or endpoint) is, how it will be compared between treatments, when it will be measured and, importantly, how post-randomisation events such as the adjustment of background therapy will be handled in the analysis. The first three attributes are very familiar to many researchers and are not discussed further. However, why do post-

For peer review only

randomisation events matter? They matter because adjustment of background medication along with other post-randomisation events such as study medication discontinuation due to an adverse event can markedly influence the treatment effect being estimated, which in turn impacts the sample size required to detect this effect and ultimately the interpretation of the study results. For instance, in PD, background medication would be changed if the patient is not deriving adequate benefit from study treatment or if the patient is experiencing dyskinesia (involuntary movements) in a clinical trial. The treating physician would normally seek to improve the patient's condition by modifying the dose of levodopa, modifying the time and frequency of administration, or adding in a new drug. From this point onwards, the patient is on a different journey from someone who continues on stable background medication, as the benefit they are deriving is affected by the change to background medication.

The researcher must decide in what situation Drug X would be considered *superior* to placebo. Is it when the treatment effect in all patients is considered *regardless* of whether their background medication was changed, or is the focus on the *hypothetical* scenario where no patient had their background medication changed? Or some intermediate scenario? A number of such scenarios are potentially of interest, but as we will see later, can yield quite different treatment effects, of differing variability, and hence impact power and sample size. Hence, the importance of clearly specifying the primary clinical question and treatment effect of interest in the protocol, which is where the estimand framework is invaluable.

The estimand framework

The ICH E9 (R1) Addendum on Estimands and Sensitivity Analysis in Clinical Trials describes a systematic approach to thinking through the trial objectives to ensure that the study goals are both precise and transparent and that the proposed design and analysis is aligned with them[2,3]. The addendum reinforces the importance of *a priori* defining the *estimand* of interest. This is a precise definition of the treatment effect we would like to estimate: a key part of this definition includes specifying how post-randomisation events (referred to as *intercurrent* events in the guideline) are handled. Based on the chosen estimand, a suitable trial design and analytical approach is chosen to ensure the trial can address the study objectives. The key aspects of the estimand framework and a schematic for common strategies for handling post-randomisation events are given in Table 1.

For peer review only

Table1: Estimands in a nutshell

- Defining an estimand entails defining five attributes: treatment condition(s), population, outcome, population-level summary and strategy for post-randomisation events. In particular it requires careful specification of how post-randomisation events such as treatment modification/discontinuation or use of rescue medication are to be handled.
- The ICH E9 (R1) addendum suggests five strategies to address post-randomisation events:
    1. Treatment policy strategy: regardless of any post-randomisation events, the treatment effect is described from the final outcome measure in all patients. Note that this approach cannot be used for truncated events, e.g. where a variable cannot be measured due to death.
    2. Hypothetical strategy: the treatment effect under a scenario where the post-randomisation event(s) did not occur.
    3. Composite strategy: all post-randomisation events are incorporated into the outcome definition, for instance, rescued patients are classed as a non-responder for a binary responder/non-responder outcome.
    4. While on treatment strategy: response to treatment before occurrence of post-randomisation event(s) is of interest.
    5. Principal stratum: the treatment effect in the "principal stratum" of interest (e.g. the population of patients who would not need rescue.
- Of course, as recognised by ICH E9 (R1), we may well wish to handle different post-randomisation events by different strategies within a study, e.g. different strategies for treatment discontinuation due to AE versus lack of efficacy.

For peer review only

As the ICH E9 (R1) guidance has been discussed in detail elsewhere[4-12], our focus is on explaining — through contrasting examples — how researchers can effectively use estimands in their trial planning, design and analysis process to ensure clinical questions are precisely specified and the chance of a successful trial is maximised. In so doing, we hope to convert readers from estimand sceptics to enthusiasts. We begin with a PD example to illustrate the lack of transparency in current study designs, and how the estimand framework can resolve this.

Example in advanced PD

To demonstrate the importance of clearly defining *a priori what* the research question is, we use information from two typical Phase 3 trials in patients with idiopathic PD with "wearing off" phenomenon[13,14]. Both studies have similar design.

During the trial patients receive either experimental or placebo treatment in addition to background therapy. A short titration/adjustment phase is followed by a maintenance phase,

during which study treatment is kept stable. The primary efficacy endpoint is the change from baseline to end of double-blind maintenance phase in absolute OFF-time. OFF periods are where PD symptoms (e.g. tremor, slowness, stiffness, walking/balance problems) reappear or worsen. The most common post-randomisation event here is adjustment of background medication to control PD symptoms or to manage dyskinesia, a complication of long-term levodopa use.

What is the estimand?

Neither study defined an estimand using ICH E9 (R1) terminology, so we first translated the available information into estimand attributes using the approach proposed by Mitroiu *et al* (which involved a degree of subjective judgment)[15]. The primary analysis was performed on what was described as the Intention-to-Treat (ITT) population, which consisted of all patients who received one dose of medication and had one post baseline OFF-time efficacy outcome assessment during the maintenance phase. To illustrate how estimands work in practice, it will be assumed that endpoints after the first post-randomisation event of change in background medication were not collected, and were instead replaced by the 'last available outcome measure' prior to the change in background medication (i.e. Last Observation Carried Forward (LOCF)). This approach to the change in background medication and discontinuation of patients is deployed in many PD studies, although it is not explicitly clear that the approach was used in either of the Phase 3 trials[13,14].

The description of Intention-to-Treat in many PD trials usually implies that the treatment effect to be estimated will correspond to the use of the treatment as introduced into routine clinical practice (i.e. the effect of a treatment policy). However, many PD trials actually estimate quite a different treatment effect, which on first glance is not readily apparent:

1. Contrary to the classic definition of ITT, patients who did not receive any study drug are excluded from the analysis, implying the intended treatment effect is either hypothetical (the effect if all patients *had* received study drug) or principal stratum (the effect in the subset of patients who *would* have received study drug under either treatment), though it is not clear from the description which of these the investigators are interested in.

For peer review only

2. Outcome data after the patient had their background medication adjusted is often set to missing and replaced using LOCF. This implies a hypothetical treatment effect (the treatment effect if adjustment of background medication had occurred).

In passing, note that we distinguish the estimand from the analysis method used to handle missing data (which should be consistent with the estimand). The estimand enables the researcher to determine what data they need to collect. If this data cannot be collected for whatever reason, then this is a missing data problem to be addressed in the statistical analysis, consistent with the estimand. In this case, using LOCF to impute outcome data after the post-randomisation event is not consistent with the ITT estimand.

In summary, a careful examination of PD trials shows that the treatment effect to be estimated is not precisely and transparently defined. Without this being stated it isn't viable to assess whether their design and analysis are appropriate. The studies are often not able (without bringing in untestable assumptions) to obtain a goal of estimating the ITT effect, if this was indeed the intention. The advantage of connecting the study objectives to the estimand upfront in the protocol removes uncertainty about the nature of the treatment effect being estimated, but also focusses attention early in the planning process how to handle post-randomisation events and what consequences this has for study design, conduct and interpretation.

An alternative estimand

Using the ICH E9 (R1) estimands framework (Table 2) we can define a treatment policy estimand, which more closely aligns with the ITT treatment effect, and which requires much weaker assumptions for estimation. Under this treatment policy estimand, OFF-times should be collected on patients after the occurrence of the post-randomisation event, and all data from all patients should be included in the analysis. Table 2 presents the assumptions of this revised Treatment Policy estimand, together with the typical estimand for PD trials.

For peer review only

162 **Table 2**: Two estimand strategies for Parkinson's Disease (PD) trials

Attribute	Typical PD trial estimand	Alternative Treatment Policy estimand
Treatment	Experimental treatment or placebo added to background therapy with adjustment of the background medication permitted during the first titration period, but not thereafter.	Experimental treatment or placebo added to background therapy, dosed as required, and alternative medication, dosed as required.
Population	Idiopathic PD with "wearing off" phenomenon	Idiopathic PD with "wearing off" phenomenon
Outcome	Change from baseline in absolute OFF-time up to the end of maintenance phase	Change from baseline in absolute OFF-time up to the end of maintenance phase
Population-level summary	Mean difference	Mean difference
Post-randomisation event	Adjustment of PD medication during the maintenance period.<br><br>Hypothetical strategy; i.e. the treatment effect in the hypothetical case where adjustment to PD medication did not occur	Adjustment of PD medication during the maintenance period.<br><br>Treatment policy strategy where data after the post randomisation events is collected and used in the analysis.

163

164 *The two estimands compared*

165 Using the information from the Phase 3 trials, we simulated OFF-times to resemble typical
166 studies for PD. To mimic the treatment policy strategy we assumed that patients with OFF-
167 times above the 90th percentile were poor responders and would have their background
168 medication changed, after which their OFF-times would improve. All OFF-times above the
169 90th percentile were replaced with randomly selected values from a normal distribution
170 reflecting a treatment from which the patient was likely to derive benefit.

171 Five hundred (500) patients were randomised in the simulation to experimental or placebo
172 treatment. Thirty-nine (39) patients in the placebo and 11 patients in the experimental arm
173 required change of background medication, because their OFF-time went above the 90th
174 percentile. The table below shows the results of the simulation.

175

176

177

For peer review only

Table 3: PD Simulation results

	Typical PD trial estimand		Alternative Treatment Policy estimand	
	Placebo	Experimental	Placebo	Experimental
Primary endpoint OFF time (hours), n	250	250	250	250
Mean (SE)	5.28 (0.10)	4.14 (0.10)	4.90 (0.08)	4.03 (0.08)
Mean difference (SE) experimental vs. PBO	-1.14 (0.14)		-0.87 (0.12)	
95% CI	(-1.43, - 0.86)		(-1.11, -0.64)	
P-value	<0.001		<0.001	

PBO: placebo; SE: standard error; CI: confidence interval

Table 3 shows the treatment effect for the treatment policy estimand (-0.87 [-1.11, -0.64]) is markedly smaller than for the typical PD trial estimand (-1.14 [-1.43, -0.86]), although both are statistically significant (p<0.001). Under treatment policy more placebo (39) than experimental (11) patients benefited from adjustment of background medication. Thus, the treatment effect being estimated changes from one strictly comparing the experimental treatment against placebo with no change to background medication in either group to one including changes to background medication in either group (realising that changes may be more common in the less effective group). Subsequently the original analysis therefore gives an inflated answer to the treatment policy question.

Which strategy should be chosen?

It depends on the main clinical question of interest. Do we want the best estimate of the effect of experimental treatment, uninfluenced by changes to background medication? This is reasonable, but should not be confused with the treatment policy question. Since typically more patients on the placebo arm are likely to require a change to background medication, for the treatment policy question the comparator isn't "placebo", but a mixture of placebo and change in background medication. Or we may be more interested in something else? Stakeholders will have different views. What is clearly important is that the researcher describes the treatment effect(s) they wish to estimate in a clear and transparent way in

For peer review only

protocols and trial reports, in particular ensuring that post-randomisation events are handled consistently with this. Then stakeholders can easily and reliably interpret the trial's findings – the ultimate goal of the using the estimands framework.

We now review a number of strategies.

Treatment policy

This is the focus if the objective is to determine the effectiveness of Drug X when used as part of an existing treatment algorithm. For instance, in the Parkinson's example, this would mean allowing clinicians to change background therapy during the study. Treatment would be specified as Drug X added to background therapy Y, dosed as required, and with additional medication, as required.

The main implication of this approach for the trial's design and conduct is that all post-randomisation data are collected, which are subsequently used in the statistical analysis. That is, all patients need to be followed up for outcome regardless of post-randomisation events. The other main implication is that the treatment effect may be smaller due to the occurrence of post-randomisation, which may increase the required sample size. In the case of the Parkinson' disease example, the reason for this is the comparator arm is not a true placebo but placebo plus change in background medication for those patients who do not perform well.

Hypothetical situation where post-randomisation event does not occur

The clinical question of interest is, what is the effect of Drug X where adjustment to background medication does not occur, regardless of any deterioration of the patient. This strategy seems to be favoured by regulatory agencies in studies with PD, as they want to distinguish between the effect of Drug X and an increased efficacy due to adjustment of levodopa[16].

The analysis methods aligned to this estimand though often requires strong assumptions about what would have happened if patients had actually continued treatment uninterrupted. Such assumptions are often unverifiable. For instance, in the PD example, the assumption was that the OFF-time would remain unchanged until the end of the observation period, which is very unlikely. Although this approach is still accepted by regulatory agencies in some clinical settings, it remains controversial[17].

For peer review only

Other approaches?

One other possibility is the composite outcome strategy, which involves re-defining the outcome to include the post-randomisation event as part of the outcome. For instance, an OFF-time responder endpoint could be defined, e.g., 1 hour or more reduction in absolute OFF-time from baseline to endpoint. Patients whose background medication changed could be classified as non-responders.

Including the post-randomisation event in the outcome definition greatly simplifies matters. However, by changing from a continuous measure (change in OFF-time) to a binary measure (reduction in OFF-time of 1 hour from baseline) alters the study question being addressed, and will increase the sample size required to address the question. Further, it is critical to precisely define "non-responder" so that we do not fundamentally change the clinical question we are asking, even though the treatment effect of interest does change; for instance, if "non-responder" is broadly defined then this would include symptom control and tolerability as well as changes to background medication. Hence, instead of looking solely at the effect of treatment on symptom control, we could also be looking at symptom control and comparative tolerability. Again, if this is the clinical question of interest, then it is fine, though that would require careful consideration by relevant stakeholders. Critically, it is essential that the target of estimation for a particular trial objective aligns with a relevant clinical research question and that this is clearly defined so that stakeholders can judge its merits: a useful composite must have clear clinical relevance.

Real-life setting

The above example has been kept simple to illustrate the basic concept of estimands. However, in real life more than one type of post-randomisation event will occur in a study, e.g. use of rescue medication, change of background medication, switching from placebo to experimental, study drug discontinuation due to an adverse event, etc.

In PD, background therapy can be changed for many reasons, most commonly motor fluctuations (wearing off) and motor complications (dyskinesia). Should we use the same or different strategies to deal with these events? In the first case background medication is adjusted to manage symptoms of the disease, which we are measuring with our outcome variable. In the second case, it is adjusted to manage an adverse event. We could therefore envisage a hypothetical strategy for motor fluctuations and a treatment policy strategy for motor complications.

For peer review only

Sometimes it may be useful to define multiple estimands within a clinical trial, if different treatment effects are of interest. If so, then researchers must specify, which estimand is the primary and which are secondary.

Case Study: Trial in advanced cancer

To illustrate application of the estimand framework in practice, consider a typical trial in advanced cancer.

Study objective

In many oncology studies, the protocol has a vague objectives statement such as "The primary objective of the study is to compare overall survival of subjects treated with experimental therapy relative to those treated with protocol-specific physician's choice". However, what does this mean in terms of the treatment effect to be estimated?

Outline study design

Patients are typically randomised to receive experimental therapy plus standard of care or placebo plus standard of care. A common design choice includes allowing placebo patients switching to experimental therapy after disease progression, even though this would not typically occur in practice. In reality patients would switch to another therapy, whenever possible. In the trial setting, patients are then followed up until death or some pre- specified time when a pre-determined number of deaths have occurred.

TransCelerate Protocol Template

TransCelerate BioPharma Inc. is an organization with a mission to drive the efficient, effective and high-quality delivery of new medicines. One area of focus was the development of a standard template for protocols, which included estimands[18]. In order to precisely understand the treatment effect being estimated we propose that the TransCelerate protocol template pertaining to estimands is adopted; namely for the oncology study:

**Objective**	**Estimand**
Primary	
The primary objective of the study is to compare overall survival of subjects treated with experimental therapy	*Treatment:* Experimental/Placebo+ Experimental on progression at discretion of local investigator<br>*Population:* "Advanced cancer"

For peer review only - http://bmjopen.bmj.com/site/about/guidelines.xhtml

relative to those treated with protocol-specific physician's choice	*Variable:* Time to death<br>*Population summary level:* Hazard ratio<br>*Post Randomisation Event*<br>• Treatment Switching for patients who progress in the control group, to be handled by a Treatment Policy strategy

The proposed estimand section documents the 5 attributes needed to understand the treatment effect of interest for a given trial. For simplicity, it has been assumed that there is a single post-randomisation event of switching to experimental treatment after disease progression. Post switching data are collected and included in the analysis as per the Treatment Policy strategy, but what does this mean in terms of the treatment effect being estimated and can causal effects be determined? After switching, placebo patients can be assigned to experimental therapy at the discretion of the local investigator, so the treatment effect being estimated equates to experimental therapy vs placebo combined with experimental therapy for many patients.

An alternative approach which leads to a more direct causal effect would be to consider a hypothetical strategy for the post randomisation event of switching. Table 4 below summarises this alternative estimand.

Table 4: TransCelerate Estimand Template

**Objective**	**Estimand**	**Alternative Estimand**
Primary		
The primary objective of the study is to compare overall survival of subjects treated with experimental therapy relative to those treated with protocol-specific physician's choice	*Treatment:*<br>Experimental/Placebo+ Experimental on progression at discretion of local investigator<br>*Population:* "Advanced cancer"<br>*Variable:* Time to death<br>*Population summary level*:<br>Hazard ratio<br>*Post Randomisation Event*	*Treatment:*<br>Experimental/Placebo<br><br>*Population:* Advanced cancer"<br>*Variable:* Time to death<br>*Population summary level:*<br>Hazard ratio<br>*Post Randomisation Event*

For peer review only

	• Treatment Switching for patients who progress in the control group, to be handled using a Treatment Policy strategy	• Treatment Switching for patients who progress in the control group to be handled by a Hypothetical strategy (i.e. the treatment effect if participants did not switch to experimental after progression)

The alternative estimation method of analysis aligned to the hypothetical estimand is inverse probability of censoring weighting (IPCW)[19]. IPCW allows us to assess the clinical benefit of the experimental arm compared with the control, by recreating an unbiased scenario where no placebo patient switched to experimental treatment[20].

What is the impact?

To illustrate the different estimands under consideration, data were generated to resemble a trial comparing an experimental anticancer treatment with placebo in advanced cancer akin to Latimer et al [21]. The data comprised the randomised group, time of switching, time of event/censoring and event indicator. Two-hundred and twenty-three (223) patients were randomised (115 to experimental, 108 to placebo) with 49 switching from placebo to experimental treatment. The results (Figure 1) show that the treatment-policy and hypothetical survival curves are very similar for the first 3 months and then diverge, with the hypothetical curve showing greater benefit. As a result, the estimated hazard ratio changes from 0.79 for the treatment-policy estimand to 0.62 for the hypothetical estimand (Table 5). The hypothetical estimand is statistically significant whereas the treatment-policy estimand is not. This likely reflects the fact that a high proportion of placebo patients switched to experimental treatment (49/108 [45%]).

For peer review only

Table 5: Oncology Study results

	Treatment Policy		Hypothetical	
	Placebo	Experimental	Placebo	Experimental
Number of Patients	115	108	115	108
Number who switch	49		49	
HR	0.79		0.62	
95% CI	(0.60, 1.04)		(0.43, 0.88)	
P-value	>0.05		<0.05	

CI: confidence interval

Which strategy should be chosen?

The actual estimand of interest varies by stakeholder. It can be argued that the hypothetical strategy provides a better causal estimate of the treatment effect. However, this estimate does depend on the methodology used and the underlying untestable assumptions. For instance, IPCW requires measurement of prognostic factors that predict treatment switching and can be prone to error when switching proportions are high[20]. The treatment policy strategy on the other hand has the advantage of including follow-up data on all patients in the analysis, which may be more directly relevant to patients —although interpretation of this comparison depends on how closely study treatment reflects clinical practice[22]. Furthermore, a high switching proportion reduces the effect size, meaning a larger sample size may be needed under the treatment policy strategy (although use of IPCW as for the hypothetical strategy can also increase the required sample size

Regardless of the strategy used, it is imperative to *a priori* precisely and transparently define the treatment effect to be estimated via estimands. The TransCelerate protocol template approach now includes the estimand attributes, which has resulted in greater transparency and clarity regarding study objectives. As illustrated, clinical trial results can be simulated to illustrate the impact of the different options for post randomisation events to stakeholders.

Discussion

The examples presented — although relatively straightforward — demonstrate that both (a) using the estimands framework brings much-needed clarity and transparency to the design and analysis of clinical trials, and (b) the choice of estimand has a clinically relevant impact on the trial results. Using estimands results in clearer answers to well specified questions, and

For peer review only

so in turn improves regulatory and clinical decision-making. Estimands also facilitate meta-analysis, potentially alleviating an important source of heterogeneity.

In the light of this, we now use estimands routinely when planning our studies. This gives invaluable guidance to choosing which data needs to be collected to answer the clinical question of interest. Inevitably this entails planning for a number of different post-randomisation events. Because handling of post-randomisation events in the analysis can require untestable assumptions, it is important to plan for and perform sensitivity analyses (consistent with the estimand) to test the robustness of the conclusions to other clinical plausible assumptions.

In conclusion, estimands are not just a talking point for statisticians. Rather, they are a key practical tool for the whole trial team, to ensure clinical questions, design, and analysis remain focussed and aligned. Following our own practice, we therefore advocate their use for all trials, and argue that they should be a mandatory part of published trial protocols.

Footnotes

Acknowledgement: We thank Nicholas R Latimer for providing the data that were used in the second simulation study.

Contributors: TPC, JRC, AP and BCK initiated the project. The first draft of the manuscript was written by TPC and AP and was refined by JRC, IRW and BCK. TPC and IRW conducted the simulations. All authors contributed to the manuscript and read and approved the final manuscript.

Funding: JC, IW and BK are funded by the UK MRC, grants MC_UU_12023/21 and MC_UU_12023/29.

Competing interests: We have read and understood the BMJ Group policy on declaration of interests and declare the following interests: TC and AP are employed by the clinical research organisation ICON.

Patient and Public Involvement: It was not appropriate or possible to involve patients or the public in the design, or conduct, or reporting, or dissemination plans of our research.

For peer review only

Data sharing statement: not relevant.

References

1. DeMaagd, George, and Ashok Philip. Parkinson's Disease and Its Management: Part 4: Treatment of Motor Complications. *P & T: a peer-reviewed journal for formulary management*, 2015; 40: 747-73.
2. ICH E9 Expert Working Group. Statistical principles for clinical trials: ICM Harmonized Tripartite Guideline. *Stat Med*, 1999; 18:1905–1942
3. Akacha M. Estimands and their role in clinical trials. Talk given at the Joint DIA/AEMPS Statistics Workshop on Statistical methodology in Clinical R&D, 2014; 10-11 November 2014, Barcelona, Spain
4. Akacha M, Bretz F, Ohlssen D, Rosenkranz G, Schmidli H. Estimands and Their Role in Clinical Trials. *Stat Bioph Res*, 2017; 9:268-71
5. Permutt T A. Defining treatment effects: A regulatory perspective. *Clinical Trials*, 2019; 16: 345–349
6. Akacha M, Bretz F, Ruberg S. Estimands in clinical trials - broadening the perspective. *Stat Med*, 2017; 36:5-19
7. Holzhauer B, Akacha M, Bermann G. Choice of Estimand and Analysis Methods in Diabetes Trials with Rescue Medication. *Pharm Stat*, 2015; 14:433-47
8. Leuchs A, Zinserling J, Brandt A, Wirtz D, Benda N. Choosing Appropriate Estimands in Clinical Trials. *TIRS*, 2015; 49:584-92
9. Permutt T A. Taxonomy of Estimands for Regulatory Clinical Trials with Discontinuations. *Stat Med*, 2016; 35:2865-75
10. Phillips A, Abellan-Andres J, Soren A, Bretz F, Fletcher C, France L, Garrett A, Harris R, Kjaer M, Keene O, Morgan D, O'Kelly M, Roger J. Estimands: discussion points from the PSI estimands and sensitivity expert group. *Pharm Stat*, 2017; 16:6-11
11. Phillips A, Clark T. Estimands in practice: Bridging the gap between study objectives and statistical analysis. *Pharm Stat*, 2021; 20: 68-76
12. Kahan BC, Morris TP, White IR, Tweed CD, Cro S, Dahly D, My Pham TM, Esmail HE, Babiker A, Carpenter J (2020) Treatment estimands in clinical trials of patients hospitalised for COVID-19: ensuring trials ask the right question. Available at https://bmcmedicine.biomedcentral.com/articles/10.1186/s12916-020-01737-0. Last accessed March 2021.
13. Ongentys™ FDA Approval Package, 2019

For peer review only

14. Neupro FDA Approval Package, 2012

15. Mitroiu, M., Oude Rengerink, K., Teerenstra, S. et al. A narrative review of estimands in drug development and regulatory evaluation: old wine in new barrels?. *Trials* 21, 671 (2020). https://doi.org/10.1186/s13063-020-04546-1

16. CHMP Guideline on clinical investigation of medicinal products in the treatment of Parkinson's disease. EMA/CHMP/330418/2012 rev. 2. June 2012

17. Lachin JM. Fallacies of last observation carried forward analyses. *Clin Trials*. 2016;13(2):161-168. doi:10.1177/1740774515602688

18. TransCelerate Biopharma Inc. Available at https://www.transceleratebiopharmainc.com/ Last accessed March 2021.

19. Williamson EJ, Forbes A, White IR. Variance reduction in randomised trials by inverse probability weighting using the propensity score. *Stat Med.* 2014;33:721–37

20. Skaltsa, K., Ivanescu, C., Naidoo, S. et al. Adjusting Overall Survival Estimates after Treatment Switching: a Case Study in Metastatic Castration-Resistant Prostate Cancer. *Targ Oncol* 12, 111–121 (2017). https://doi.org/10.1007/s11523-016-0472-3

21. Latimer NR, Abrams KR, Lambert PC, Crowther MJ, Wailoo AJ, Morden JP, Akenhurst RL, Campbell MJ (2017) Adjusting for treatment switching in randomised controlled trials – A simulation study and a simplified two-stage method. Stat Methods Med Res 2017;26:724–51. Available from: http://smm.sagepub.com/cgi/doi/10.1177/0962280214557578

22. Degtyarev E, Zhang Y, Sen K, Lebwohl D, Akacha M, Hampson LV, Bornkamp B, Maniero A, Bretz F, and Zuber E. Estimands and the Patient Journey: Addressing the Right Question in Oncology Clinical Trials. *JCO Precision Oncology* 2019 :3, 1-10

For peer review only

462 **Figure 1: Kaplan-Meir Survival Plots for Oncology Study**

For peer review only

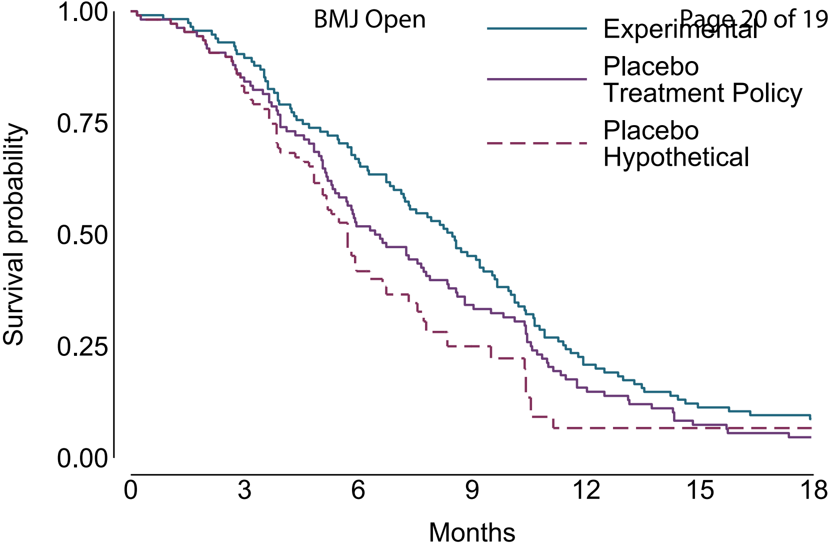

Number at risk

	0	3	6	9	12	15	18
Experimental	115	103	77	52	24	13	0
Placebo Treatment Policy	108	91	56	37	17	8	0
Placebo Hypothetical	108	84	26	11	3	3	0

For peer review only - http://bmjopen.bmj.com/site/about/guidelines.xhtml

BMJ Open

Estimands: Bringing clarity and focus to research questions in clinical trials

Journal:	*BMJ Open*
Manuscript ID	bmjopen-2021-052953.R2
Article Type:	Communication
Date Submitted by the Author:	12-Oct-2021

Complete List of Authors:	Clark, Timothy; MRC Clinical Trials Unit at UCL, Institute of Clinical Trials & Methodology<br>Kahan, Brennan; MRC Clinical Trials Unit at UCL<br>Phillips, Alan; ICON Clinical Research UK Ltd, Biostatistics;<br>White, Ian; MRC Clinical Trials Unit at UCL, Clinical Trials & Methodology<br>Carpenter, James; MRC Clinical Trials Unit at UCL, London Hub for Trials Methodology Research; London School of Hygiene & Tropical Medicine, Department of Medical Statistics

**Primary Subject Heading**:	Research methods
Secondary Subject Heading:	Communication, Medical education and training, Medical publishing and peer review
Keywords:	Protocols & guidelines < HEALTH SERVICES ADMINISTRATION & MANAGEMENT, MEDICAL EDUCATION & TRAINING, QUALITATIVE RESEARCH

SCHOLARONE™
Manuscripts

I, the Submitting Author has the right to grant and does grant on behalf of all authors of the Work (as defined in the below author licence), an exclusive licence and/or a non-exclusive licence for contributions from authors who are: i) UK Crown employees; ii) where BMJ has agreed a CC-BY licence shall apply, and/or iii) in accordance with the terms applicable for US Federal Government officers or employees acting as part of their official duties; on a worldwide, perpetual, irrevocable, royalty-free basis to BMJ Publishing Group Ltd ("BMJ") its licensees and where the relevant Journal is co-owned by BMJ to the co-owners of the Journal, to publish the Work in BMJ Open and any other BMJ products and to exploit all rights, as set out in our licence.

The Submitting Author accepts and understands that any supply made under these terms is made by BMJ to the Submitting Author unless you are acting as an employee on behalf of your employer or a postgraduate student of an affiliated institution which is paying any applicable article publishing charge ("APC") for Open Access articles. Where the Submitting Author wishes to make the Work available on an Open Access basis (and intends to pay the relevant APC), the terms of reuse of such Open Access shall be governed by a Creative Commons licence – details of these licences and which Creative Commons licence will apply to this Work are set out in our licence referred to above.

Other than as permitted in any relevant BMJ Author's Self Archiving Policies, I confirm this Work has not been accepted for publication elsewhere, is not being considered for publication elsewhere and does not duplicate material already published. I confirm all authors consent to publication of this Work and authorise the granting of this licence.

For peer review only

Communication

Estimands: Bringing clarity and focus to research questions in clinical trials

Timothy P Clark, honorary associate professor of medical statistics[1], Brennan C Kahan, senior research fellow[1], Alan Phillips, Sr Director, Biostatistics[2], Ian R White, professor of statistical methods for medicine[1], James R Carpenter, professor of medical statistics[1]

Correspondence to: T Clark, **MRC Clinical Trials Unit at UCL,** Institute of Clinical Trials & Methodology, 90 High Holborn, 2nd Floor, London WC1V 6LJ
Tel: +44 (0)20 7670-4700
E-mail: tim.clark@ucl.ac.uk

Keywords: estimands; estimation; research question; treatment effect; post-randomisation events.

Word count: 3,808

[1]MRC Clinical Trials Unit at UCL, Institute of Clinical Trials andMethodology, University College London, London, UK
[2]Innovation Center, ICON Clinical Research, Marlow, UK

For peer review only

Abstract

Precise specification of the research question and associated treatment effect of interest is essential in clinical research, yet recent work shows that they are often incompletely specified. The ICH E9 (R1) Addendum on Estimands and Sensitivity Analysis in Clinical Trials introduces a framework that supports researchers in precisely and transparently specifying the treatment effect they aim to estimate in their clinical trial. In this paper we present practical examples to demonstrate to all researchers involved in clinical trials how estimands can help them to specify the research question, lead to a better understanding of the treatment effect to be estimated and hence increase the probability of success of the trial.

Background

To illustrate the need for estimands, consider some typical wording for the primary objective of a Parkinson's disease (PD) clinical study:

The primary objective of this study is to demonstrate that Drug X is superior to placebo in patients with idiopathic Parkinson's disease with "wearing off" phenomenon.

We are all familiar with such statements, but what do they actually tell us about the treatment effect to be estimated? We know the treatments to be compared, the patient population of interest and that the aim of the study is to show superiority. An experienced researcher in the field would further recognise that these are patients with advanced PD who are currently receiving levodopa and potentially other medications such as dopamine agonists; that these patients have wearing off phenomenon, which means that the effect of levodopa is wearing off between doses, i.e., Parkinson's symptoms such as problems with movement (motor symptoms) return; and finally, that the study is being conducted to see if the addition of Drug X to background medication consisting of levodopa plus another anti-Parkinson drug, e.g. dopamine agonist or MAO-B inhibitor, will improve control of motor symptoms[1].

Post-randomisation events

To fully understand the treatment effect of interest, the objective statement additionally needs to convey what the outcome variable (or endpoint) is, how it will be compared between treatments, when it will be measured and, importantly, how post-randomisation events such as the adjustment of background therapy will be handled in the analysis. The first three attributes are very familiar to many researchers and are not discussed further. However, why do post-

For peer review only

randomisation events matter? They matter because adjustment of background medication along with other post-randomisation events such as study medication discontinuation due to an adverse event can markedly influence the treatment effect being estimated, which in turn impacts the sample size required to detect this effect and ultimately the interpretation of the study results. For instance, in PD, background medication would be changed if the patient is not deriving adequate benefit from study treatment or if the patient is experiencing dyskinesia (involuntary movements) in a clinical trial. The treating physician would normally seek to improve the patient's condition by modifying the dose of levodopa, modifying the time and frequency of administration, or adding in a new drug. From this point onwards, the patient is on a different journey from someone who continues on stable background medication, as the benefit they are deriving is affected by the change to background medication.

The researcher must decide in what situation Drug X would be considered *superior* to placebo. Is it when the treatment effect in all patients is considered *regardless* of whether their background medication was changed, or is the focus on the *hypothetical* scenario where no patient had their background medication changed? Or some intermediate scenario? A number of such scenarios are potentially of interest, but as we will see later, can yield quite different treatment effects, of differing variability, and hence impact power and sample size. Hence, the importance of clearly specifying the primary clinical question and treatment effect of interest in the protocol, which is where the estimand framework is invaluable.

The estimand framework

The ICH E9 (R1) Addendum on Estimands and Sensitivity Analysis in Clinical Trials describes a systematic approach to thinking through the trial objectives to ensure that the study goals are both precise and transparent and that the proposed design and analysis is aligned with them[2,3]. The addendum reinforces the importance of *a priori* defining the *estimand* of interest. This is a precise definition of the treatment effect we would like to estimate: a key part of this definition includes specifying how post-randomisation events (referred to as *intercurrent* events in the guideline) are handled. Based on the chosen estimand, a suitable trial design and analytical approach is chosen to ensure the trial can address the study objectives. The key aspects of the estimand framework and a schematic for common strategies for handling post-randomisation events are given in Table 1.

For peer review only

Table1: Estimands in a nutshell

- Defining an estimand entails defining five attributes: treatment condition(s), population, outcome, population-level summary and strategy for post-randomisation events. In particular it requires careful specification of how post-randomisation events such as treatment modification/discontinuation or use of rescue medication are to be handled.
- The ICH E9 (R1) addendum suggests five strategies to address post-randomisation events:
    1. Treatment policy strategy: regardless of any post-randomisation events, the treatment effect is described from the final outcome measure in all patients. Note that this approach cannot be used for truncated events, e.g. where a variable cannot be measured due to death.
    2. Hypothetical strategy: the treatment effect under a scenario where the post-randomisation event(s) did not occur.
    3. Composite strategy: all post-randomisation events are incorporated into the outcome definition, for instance, rescued patients are classed as a non-responder for a binary responder/non-responder outcome.
    4. While on treatment strategy: response to treatment before occurrence of post-randomisation event(s) is of interest.
    5. Principal stratum: the treatment effect in the "principal stratum" of interest (e.g. the population of patients who would not need rescue.
- Of course, as recognised by ICH E9 (R1), we may well wish to handle different post-randomisation events by different strategies within a study, e.g. different strategies for treatment discontinuation due to AE versus lack of efficacy.

As the ICH E9 (R1) guidance has been discussed in detail elsewhere[4-13], our focus is on explaining — through contrasting examples — how researchers can effectively use estimands in their trial planning, design and analysis process to ensure clinical questions are precisely specified and the chance of a successful trial is maximised. In so doing, we hope to convert readers from estimand sceptics to enthusiasts.

We continue with the PD example to illustrate the lack of transparency in current study designs, and how the estimand framework can resolve this. We will explain through a second example in advanced cancer how the estimand framework can be applied in practice.

Example in advanced PD

To demonstrate the importance of clearly defining *a priori what* the research question is, we use information from two typical Phase 3 trials in patients with idiopathic PD with "wearing off" phenomenon[14,15]. Both studies have similar design.

For peer review only

During the trial patients receive either experimental or placebo treatment in addition to background therapy. A short titration/adjustment phase is followed by a maintenance phase, during which study treatment is kept stable. The primary efficacy endpoint is the change from baseline to end of double-blind maintenance phase in absolute OFF-time. OFF periods are where PD symptoms (e.g. tremor, slowness, stiffness, walking/balance problems) reappear or worsen. The most common post-randomisation event here is adjustment of background medication to control PD symptoms or to manage dyskinesia, a complication of long-term levodopa use.

What is the estimand?

Neither of the Phase 3 trials in patients with idiopathic PD defined an estimand using ICH E9 (R1) terminology, so we first translated the available information into estimand attributes using the approach proposed by Mitroiu *et al* (which involved a degree of subjective judgment)[16]. The primary analysis was performed on what was described as the Intention-to-Treat (ITT) population, which consisted of all patients who received one dose of medication and had one post baseline OFF-time efficacy outcome assessment during the maintenance phase. To illustrate how estimands work in practice, it will be assumed that endpoints after the first post-randomisation event of change in background medication were not collected, and were instead replaced by the 'last available outcome measure' prior to the change in background medication (i.e. Last Observation Carried Forward (LOCF)). This approach to the change in background medication and discontinuation of patients is deployed in many PD studies, although it is not explicitly clear that the approach was used in either of the Phase 3 trials[14,15].

The description of Intention-to-Treat in many PD trials usually implies that the treatment effect to be estimated will correspond to the use of the treatment as introduced into routine clinical practice (i.e. the effect of a treatment policy). However, many PD trials actually estimate quite a different treatment effect, which on first glance is not readily apparent:

1. Contrary to the classic definition of ITT, patients who did not receive any study drug are excluded from the analysis, implying the intended treatment effect is either hypothetical (the effect if all patients *had* received study drug) or principal stratum (the effect in the subset of patients who *would* have received study drug under either treatment), though it is not clear from the description which of these the investigators are interested in.

For peer review only

2. Outcome data after the patient had their background medication adjusted is often set to missing and replaced using LOCF. This implies a hypothetical treatment effect (the treatment effect if adjustment of background medication had not occurred).

In passing, note that we distinguish the estimand from the analysis method used to handle missing data (which should be consistent with the estimand). The estimand enables the researcher to determine what data they need to collect. If this data cannot be collected for whatever reason, then this is a missing data problem to be addressed in the statistical analysis, consistent with the estimand. In this case, using LOCF to impute outcome data after the post-randomisation event is not consistent with the ITT estimand.

In summary, a careful examination of PD trials shows that the treatment effect to be estimated is not precisely and transparently defined. Without this being stated it isn't viable to assess whether their design and analysis are appropriate. The studies are often not able (without bringing in untestable assumptions) to obtain a goal of estimating the ITT effect, if this was indeed the intention. The advantage of connecting the study objectives to the estimand upfront in the protocol removes uncertainty about the nature of the treatment effect being estimated, but also focusses attention early in the planning process how to handle post-randomisation events and what consequences this has for study design, conduct and interpretation.

An alternative estimand

Using the ICH E9 (R1) estimands framework (Table 2) we can define a treatment policy estimand, which more closely aligns with the ITT treatment effect, and which requires much weaker assumptions for estimation. Under this treatment policy estimand, OFF-times should be collected on patients after the occurrence of the post-randomisation event, and all data from all patients should be included in the analysis. Table 2 presents the assumptions of this revised treatment policy estimand, together with the typical estimand for PD trials.

For peer review only

Table 2: Two estimand strategies for Parkinson's Disease (PD) trials

Attribute	Typical PD Trial Estimand	Alternative Treatment Policy Estimand
Treatment	Experimental treatment or placebo added to background therapy with adjustment of the background medication permitted during the first titration period, but not thereafter.	Experimental treatment or placebo added to background therapy, dosed as required, and alternative medication, dosed as required.
Population	Idiopathic PD with "wearing off" phenomenon	Idiopathic PD with "wearing off" phenomenon
Outcome	Change from baseline in absolute OFF-time up to the end of maintenance phase	Change from baseline in absolute OFF-time up to the end of maintenance phase
Population-level summary	Mean difference	Mean difference
Post-randomisation event	Adjustment of PD medication during the maintenance period.<br><br>Hypothetical strategy; i.e. the treatment effect in the hypothetical case where adjustment to PD medication did not occur	Adjustment of PD medication during the maintenance period.<br><br>Treatment policy strategy where data after the post randomisation events is collected and used in the analysis.

The two estimands compared

Using the information from the Phase 3 trials, we simulated OFF-times to resemble typical studies for PD. To mimic the treatment policy strategy we assumed that patients with OFF-times above the 90th percentile were poor responders and would have their background medication changed, after which their OFF-times would improve. All OFF-times above the 90th percentile were replaced with randomly selected values from a normal distribution reflecting a treatment from which the patient was likely to derive benefit.

Five hundred (500) patients were randomised in the simulation to experimental or placebo treatment. Thirty-nine (39) patients in the placebo and 11 patients in the experimental arm required change of background medication, because their OFF-time went above the 90th percentile. The table below shows the results of the simulation.

For peer review only

Table 3: PD Simulation results

	Typical PD Trial Estimand		Alternative Treatment Policy Estimand	
	Placebo	Experimental	Placebo	Experimental
Primary endpoint OFF time (hours), n	250	250	250	250
Mean (SE)	5.28 (0.10)	4.14 (0.10)	4.90 (0.08)	4.03 (0.08)
Mean difference (SE) experimental vs. PBO	-1.14 (0.14)		-0.87 (0.12)	
95% CI	(-1.43, - 0.86)		(-1.11, -0.64)	
P-value	<0.001		<0.001	

PBO: placebo; SE: standard error; CI: confidence interval

For peer review only

Table 3 shows the treatment effect for the treatment policy estimand (-0.87 [-1.11, -0.64]) is markedly smaller than for the typical PD trial estimand (-1.14 [-1.43, -0.86]), although both are statistically significant (p<0.001). Under treatment policy more placebo (39) than experimental (11) patients benefited from adjustment of background medication. Thus, the treatment effect being estimated changes from one strictly comparing the experimental treatment against placebo with no change to background medication in either group to one including changes to background medication in either group (realising that changes may be more common in the less effective group). Subsequently the original analysis therefore gives an inflated answer to the treatment policy question.

Which strategy for post-randomisation events should be chosen?

The strategy depends on the main clinical question of interest. Do we want the best estimate of the effect of experimental treatment, uninfluenced by changes to background medication? This is reasonable, but should not be confused with the treatment policy question. Since typically more patients on the placebo arm are likely to require a change to background medication, for the treatment policy question the comparator isn't "placebo", but a mixture of placebo and change in background medication. Or we may be more interested in something else? Stakeholders will have different views. What is clearly important is that the researcher describes the treatment effect(s) they wish to estimate in a clear and transparent way in

protocols and trial reports, in particular ensuring that post-randomisation events are handled consistently with this. Then stakeholders can easily and reliably interpret the trial's findings – the ultimate goal of the using the estimands framework.

We now review a number of strategies described in the ICH E9 (R1) estimands framework using the PD example.

Treatment policy

Under treatment policy, the objective is to determine the effectiveness of Drug X when used as part of an existing treatment algorithm. In this respect it can be seen as pragmatic, as we are comparing treatments under the conditions in which they would be used in practice[17,18]. For instance, in the Parkinson's example, this would mean allowing clinicians to change background therapy during the study. Treatment would be specified as Drug X added to background therapy Y, dosed as required, and with additional medication, as required.

The main implication of this approach for the trial's design and conduct is that all post-randomisation data are collected, which are subsequently used in the statistical analysis. That is, all patients need to be followed up for outcome regardless of post-randomisation events. The other main implication is that the treatment effect may be smaller due to the occurrence of post-randomisation, which may increase the required sample size. In the case of the Parkinson' disease example, the reason for this is the comparator arm is not a true placebo but placebo plus change in background medication for those patients who do not perform well.

Hypothetical situation where post-randomisation event does not occur

The clinical question of interest is, what is the effect of Drug X where adjustment to background medication does not occur, regardless of any deterioration of the patient. This is akin to an explanatory trial, i.e., a clinical study undertaken in an idealised setting [17,18]. This strategy seems to be favoured by regulatory agencies in studies with PD, as they want to distinguish between the effect of Drug X and an increased efficacy due to adjustment of levodopa[19].

The analysis methods aligned to this estimand though often requires strong assumptions about what would have happened if patients had actually continued treatment uninterrupted. Such assumptions are often unverifiable. For instance, in the PD example, the assumption was that

For peer review only

the OFF-time would remain unchanged until the end of the observation period, which is very unlikely. Although this approach is still accepted by regulatory agencies in some clinical settings, it remains controversial[20].

Other approaches?

One other possibility is the composite outcome strategy, which involves re-defining the outcome to include the post-randomisation event as part of the outcome. For instance, an OFF-time responder endpoint could be defined, e.g., 1 hour or more reduction in absolute OFF-time from baseline to endpoint. Patients whose background medication changed could be classified as non-responders.

Including the post-randomisation event in the outcome definition greatly simplifies matters. However, by changing from a continuous measure (change in OFF-time) to a binary measure (reduction in OFF-time of 1 hour from baseline) alters the study question being addressed, and will increase the sample size required to address the question. Further, it is critical to precisely define "non-responder" so that we do not fundamentally change the clinical question we are asking, even though the treatment effect of interest does change; for instance, if "non-responder" is broadly defined then this would include symptom control and tolerability as well as changes to background medication. Hence, instead of looking solely at the effect of treatment on symptom control, we could also be looking at symptom control and comparative tolerability. Again, if this is the clinical question of interest, then it is fine, though that would require careful consideration by relevant stakeholders. Critically, it is essential that the target of estimation for a particular trial objective aligns with a relevant clinical research question and that this is clearly defined so that stakeholders can judge its merits: a useful composite must have clear clinical relevance.

Real-life setting

The above example has been kept simple to illustrate the basic concept of estimands. However, in real life more than one type of post-randomisation event will occur in a study, e.g. use of rescue medication, change of background medication, switching from placebo to experimental, study drug discontinuation due to an adverse event, etc.

In PD, background therapy can be changed for many reasons, most commonly motor fluctuations (wearing off) and motor complications (dyskinesia). Should we use the same or different strategies to deal with these events? In the first case background medication is

For peer review only

adjusted to manage symptoms of the disease, which we are measuring with our outcome variable. In the second case, it is adjusted to manage an adverse event. We could therefore envisage a hypothetical strategy for motor fluctuations and a treatment policy strategy for motor complications.

Sometimes it may be useful to define multiple estimands within a clinical trial, if different treatment effects are of interest. If so, then researchers must specify, which estimand is the primary and which are secondary.

Case Study: Trial in advanced cancer

To illustrate application of the estimand framework in practice, consider a typical trial in advanced cancer.

Study objective

In many oncology studies, the protocol has a vague objectives statement such as "The primary objective of the study is to compare overall survival of subjects treated with experimental therapy relative to those treated with protocol-specific physician's choice". However, what does this mean in terms of the treatment effect to be estimated?

Outline study design

Patients are typically randomised to receive experimental therapy plus standard of care or placebo plus standard of care. A common design choice includes allowing placebo patients switching to experimental therapy after disease progression, even though this would not typically occur in practice. In reality patients would switch to another therapy, whenever possible. In the trial setting, patients are then followed up until death or some pre- specified time when a pre-determined number of deaths have occurred.

TransCelerate Protocol Template

TransCelerate BioPharma Inc. is an organization with a mission to drive the efficient, effective and high-quality delivery of new medicines. One area of focus was the development of a standard template for protocols, which included estimands[21]. In order to precisely understand the treatment effect being estimated we propose that the TransCelerate protocol template pertaining to estimands is adopted.

For simplicity, it has been assumed that there is a single post-randomisation event of switching to experimental treatment after disease progression. Post switching data are

For peer review only

typically collected and included in the analysis as per the treatment policy strategy, but what does this mean in terms of the treatment effect being estimated and can causal effects be determined? After switching, placebo patients can be assigned to experimental therapy at the discretion of the local investigator, so the treatment effect being estimated equates to experimental therapy vs placebo combined with experimental therapy for many patients.

An alternative approach which leads to a more direct causal effect would be to consider a hypothetical strategy for the post randomisation event of switching. Table 4 below summarises the treatment policy and the alternative hypothetical estimand.

Table 4: TransCelerate Estimand Template

**Objective**	**Treatment Policy Estimand**	**Alternative Hypothetical Estimand**
Primary		
The primary objective of the study is to compare overall survival of subjects treated with experimental therapy relative to those treated with protocol-specific physician's choice	*Treatment:*<br>Experimental/Placebo+ Experimental on progression at discretion of local investigator<br>*Population:* "Advanced cancer"<br>*Variable:* Time to death<br>*Population summary level*:<br>Hazard ratio<br>*Post Randomisation Event*<br>• Treatment Switching for patients who progress in the control group, to be handled using a treatment policy strategy	*Treatment:*<br>Experimental/Placebo<br><br>*Population:* Advanced cancer"<br>*Variable:* Time to death<br>*Population summary level:*<br>Hazard ratio<br>*Post Randomisation Event*<br>• Treatment Switching for patients who progress in the control group to be handled by a Hypothetical strategy (i.e. the treatment effect if participants did not switch to experimental after progression)

For peer review only

The alternative estimation method of analysis aligned to the hypothetical estimand is inverse probability of censoring weighting (IPCW)[22]. IPCW allows us to assess the clinical benefit of the experimental arm compared with the control, by recreating an unbiased scenario where no placebo patient switched to experimental treatment[23].

What is the impact?

To illustrate the different estimands under consideration, data were generated to resemble a trial comparing an experimental anticancer treatment with placebo in advanced cancer akin to Latimer et al [24]. The data comprised the randomised group, time of switching, time of event/censoring and event indicator. Two-hundred and twenty-three (223) patients were randomised (115 to experimental, 108 to placebo) with 49 switching from placebo to experimental treatment. The results (Figure 1) show that the treatment-policy and hypothetical survival curves are very similar for the first 3 months and then diverge, with the hypothetical curve showing greater benefit. As a result, the estimated hazard ratio changes from 0.79 for the treatment-policy estimand to 0.62 for the hypothetical estimand (Table 5). The hypothetical estimand is statistically significant whereas the treatment-policy estimand is not. This likely reflects the fact that a high proportion of placebo patients switched to experimental treatment (49/108 [45%]).

Table 5: Oncology Study results

	**Treatment Policy Estimand**		**Hypothetical Estimand**	
	Placebo	Experimental	Placebo	Experimental
Number of Patients	115	108	115	108
Number who switch	49		49	
HR	0.79		0.62	
95% CI	(0.60, 1.04)		(0.43, 0.88)	
P-value	>0.05		<0.05	

CI: confidence interval

Which strategy should be chosen?

The actual estimand of interest varies by stakeholder. It can be argued that the hypothetical strategy provides a better causal estimate of the treatment effect. However, this estimate does depend on the methodology used and the underlying untestable assumptions. For instance,

For peer review only

IPCW requires measurement of prognostic factors that predict treatment switching and can be prone to error when switching proportions are high[23]. The treatment policy strategy on the other hand has the advantage of including follow-up data on all patients in the analysis, which may be more directly relevant to patients —although interpretation of this comparison depends on how closely study treatment reflects clinical practice[25]. Furthermore, a high switching proportion reduces the effect size, meaning a larger sample size may be needed under the treatment policy strategy (although use of IPCW as for the hypothetical strategy can also increase the required sample size

Regardless of the strategy used, it is imperative to *a priori* precisely and transparently define the treatment effect to be estimated via estimands. The TransCelerate protocol template approach now includes the estimand attributes, which has resulted in greater transparency and clarity regarding study objectives. As illustrated, clinical trial results can be simulated to illustrate the impact of the different options for post randomisation events to stakeholders.

Discussion

The examples presented — although relatively straightforward — demonstrate that both (a) using the estimands framework brings much-needed clarity and transparency to the design and analysis of clinical trials, and (b) the choice of estimand has a clinically relevant impact on the trial results. Using estimands results in clearer answers to well specified questions, and so in turn improves regulatory and clinical decision-making. Estimands also facilitate meta-analysis, potentially alleviating an important source of heterogeneity.

In the light of this, we now use estimands routinely when planning our studies. This gives invaluable guidance to choosing which data needs to be collected to answer the clinical question of interest. Inevitably this entails planning for a number of different post-randomisation events. Because handling of post-randomisation events in the analysis can require untestable assumptions, it is important to plan for and perform sensitivity analyses (consistent with the estimand) to test the robustness of the conclusions to other clinical plausible assumptions.

In conclusion, estimands are not just a talking point for statisticians. Rather, they are a key practical tool for the whole trial team, to ensure clinical questions, design, and analysis remain focussed and aligned. Following our own practice, we therefore advocate their use for all trials, and argue that they should be a mandatory part of published trial protocols.

For peer review only

Footnotes

Acknowledgement: We thank Nicholas R Latimer for providing the data that were used in the second simulation study.

Contributors: TPC, JRC, AP and BCK initiated the project. The first draft of the manuscript was written by TPC and AP and was refined by JRC, IRW and BCK. TPC and IRW conducted the simulations. All authors contributed to the manuscript and read and approved the final manuscript.

Funding: JC, IW and BK are funded by the UK MRC, grants MC_UU_12023/21 and MC_UU_12023/29.

Competing interests: We have read and understood the BMJ Group policy on declaration of interests and declare the following interests: TC and AP are employed by the clinical research organisation ICON.

Patient and Public Involvement: It was not appropriate or possible to involve patients or the public in the design, or conduct, or reporting, or dissemination plans of our research.

Data sharing statement: not relevant.

References

1. DeMaagd, George, and Ashok Philip. Parkinson's Disease and Its Management: Part 4: Treatment of Motor Complications. *P & T: a peer-reviewed journal for formulary management*, 2015; 40: 747-73.
2. ICH E9 Expert Working Group. Statistical principles for clinical trials: ICM Harmonized Tripartite Guideline. *Stat Med*, 1999; 18:1905–1942
3. Akacha M. Estimands and their role in clinical trials. Talk given at the Joint DIA/AEMPS Statistics Workshop on Statistical methodology in Clinical R&D, 2014; 10-11 November 2014, Barcelona, Spain
4. Akacha M, Bretz F, Ohlssen D, Rosenkranz G, Schmidli H. Estimands and Their Role in Clinical Trials. *Stat Bioph Res*, 2017; 9:268-71
5. Permutt T A. Defining treatment effects: A regulatory perspective. *Clinical Trials*, 2019; 16: 345–349

For peer review only

6. Akacha M, Bretz F, Ruberg S. Estimands in clinical trials - broadening the perspective. *Stat Med*, 2017; 36:5-19

7. Holzhauer B, Akacha M, Bermann G. Choice of Estimand and Analysis Methods in Diabetes Trials with Rescue Medication. *Pharm Stat*, 2015; 14:433-47

8. Leuchs A, Zinserling J, Brandt A, Wirtz D, Benda N. Choosing Appropriate Estimands in Clinical Trials. *TIRS*, 2015; 49:584-92

9. Permutt T A. Taxonomy of Estimands for Regulatory Clinical Trials with Discontinuations. *Stat Med*, 2016; 35:2865-75

10. Phillips A, Abellan-Andres J, Soren A, Bretz F, Fletcher C, France L, Garrett A, Harris R, Kjaer M, Keene O, Morgan D, O'Kelly M, Roger J. Estimands: discussion points from the PSI estimands and sensitivity expert group. *Pharm Stat*, 2017; 16:6-11

11. Phillips A, Clark T. Estimands in practice: Bridging the gap between study objectives and statistical analysis. *Pharm Stat*, 2021; 20: 68-76

12. Kahan BC, Morris TP, White IR, Tweed CD, Cro S, Dahly D, My Pham TM, Esmail HE, Babiker A, Carpenter J (2020) Treatment estimands in clinical trials of patients hospitalised for COVID-19: ensuring trials ask the right question. Available at https://bmcmedicine.biomedcentral.com/articles/10.1186/s12916-020-01737-0. Last accessed March 2021.

13. Kahan, B.C., Morris, T.P., White, I.R. et al. Estimands in published protocols of randomised trials: urgent improvement needed. *Trials* 22, 686 (2021). https://doi.org/10.1186/s13063-021-05644-4

14. Ongentys™ FDA Approval Package, 2019

15. Neupro FDA Approval Package, 2012

16. Mitroiu, M., Oude Rengerink, K., Teerenstra, S. et al. A narrative review of estimands in drug development and regulatory evaluation: old wine in new barrels?. *Trials* 21, 671 (2020). https://doi.org/10.1186/s13063-020-04546-1

17. Loudon K, Treweek S, Sullivan F, Donnan P, Thorpe K E, Zwarenstein M *et al*. The PRECIS-2 tool: designing trials that are fit for purpose *BMJ* 2015; 350 :h2147 doi:10.1136/bmj.h2147

18. Schwartz D, Lellouch J. Explanatory and pragmatic attitudes in therapeutical trials. *J Clin Epidemiol. 2009* May;62(5):499-505. doi: 10.1016/j.jclinepi.2009.01.012. PMID: 19348976.

19. CHMP Guideline on clinical investigation of medicinal products in the treatment of Parkinson’s disease. EMA/CHMP/330418/2012 rev. 2. June 2012

20. Lachin JM. Fallacies of last observation carried forward analyses. *Clin Trials*. 2016;13(2):161-168. doi:10.1177/1740774515602688

21. TransCelerate Biopharma Inc. Available at https://www.transceleratebiopharmainc.com/ Last accessed March 2021.

22. Williamson EJ, Forbes A, White IR. Variance reduction in randomised trials by inverse probability weighting using the propensity score. *Stat Med.* 2014;33:721–37

23. Skaltsa, K., Ivanescu, C., Naidoo, S. et al. Adjusting Overall Survival Estimates after Treatment Switching: a Case Study in Metastatic Castration-Resistant Prostate Cancer. *Targ Oncol* 12, 111–121 (2017). https://doi.org/10.1007/s11523-016-0472-3

24. Latimer NR, Abrams KR, Lambert PC, Crowther MJ, Wailoo AJ, Morden JP, Akenhurst RL, Campbell MJ (2017) Adjusting for treatment switching in randomised controlled trials – A simulation study and a simplified two-stage method. Stat Methods Med Res 2017;26:724–51. Available from: http://smm.sagepub.com/cgi/doi/10.1177/0962280214557578

25. Degtyarev E, Zhang Y, Sen K, Lebwohl D, Akacha M, Hampson LV, Bornkamp B, Maniero A, Bretz F, and Zuber E. Estimands and the Patient Journey: Addressing the Right Question in Oncology Clinical Trials. *JCO Precision Oncology* 2019 :3, 1-10

For peer review only

469 **Figure 1: Kaplan-Meir Survival Plots for Oncology Study**

For peer review only

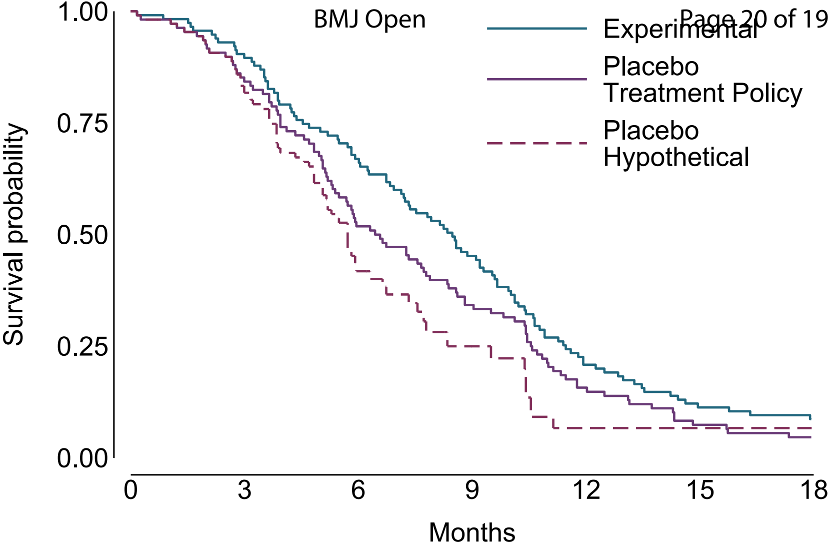

Number at risk

Experimental	115	103	77	52	24	13	0
Placebo Treatment Policy	108	91	56	37	17	8	0
Placebo Hypothetical	108	84	26	11	3	3	0

For peer review only - http://bmjopen.bmj.com/site/about/guidelines.xhtml
